# The relationship between swallowing training after total laryngectomy and the incidence of pharyngeal fistula a retrospective study with nursing-related insights

**DOI:** 10.3389/fmed.2025.1632382

**Published:** 2025-11-11

**Authors:** Xi Yang, Wenbi Jia

**Affiliations:** Department of Nursing, The First Affiliated Hospital of Chongqing Medical University, Chongqing, China

**Keywords:** total laryngectomy, pharyngeal fistula, swallowing rehabilitation, nursing intervention, postoperative complications, dysphagia management, multidisciplinary care

## Abstract

**Background:**

Pharyngeal fistula (PF), a critical complication in 10%–30% of total laryngectomy (TL) patients, delays recovery and increases healthcare costs. Current guidelines lack consensus on non-surgical prevention strategies, particularly nursing-led interventions. This study introduces an evidence-based structured swallowing training protocol integrating viscosity-modified diets, breath-holding exercises, and sensory stimulation, which was associated with reduced PF risk under systematic nursing supervision.

**Methods:**

In this single-center retrospective cohort study, 430 TL patients were enrolled: 220 received structured swallowing training initiated 10–14 days postoperatively (3×/day for 4 weeks), while 210 received standard care. The intervention comprised (1) diet progression with dry swallowing exercises, (2) seated breath-holding to trigger glottic closure, (3) tongue resistance and laryngeal elevation, and (4) sensory stimulation (preoperative taste activation, intraoperative pharyngeal brushing, postoperative imagery). Primary outcomes were PF incidence and severity; multivariate logistic regression was used to identify independent predictors (adjusted OR, 95% CI).

**Results:**

Swallowing training was associated with a 34.2% lower PF incidence (10.0% vs. 15.2%, *P* = 0.034) and fewer severe cases (68.2% mild vs. 37.5%, *P* = 0.021). Multivariate analysis confirmed training as an independent protective factor (adjusted OR = 0.55, 95% CI = 0.32–0.95, *P* = 0.031), while intraoperative blood loss ≥ 400 mL was an independent risk factor (adjusted OR = 1.75, 95% CI = 1.02–3.00, *P* = 0.043). Adherence was high (84.1%), and protocol fidelity reached 98.6%.

**Conclusion:**

Nursing-led structured swallowing training was independently associated with a 45% lower risk of PF after TL, providing a scalable and cost-effective rehabilitation framework that underscores the essential role of nursing in postoperative complication prevention.

## Introduction

Total laryngectomy (TL) is a definitive treatment for advanced laryngeal and hypopharyngeal squamous cell carcinoma, aiming to achieve oncologic control while preserving functional outcomes (1, 2). However, pharyngeal fistula (PF), a common and severe complication occurring in 10%–30% of cases, significantly impairs postoperative recovery, increases healthcare costs, and delays adjuvant therapies such as radiotherapy (3). PF arises from anastomotic dehiscence, leading to communication between the pharynx and cervical skin, and is associated with malnutrition, aspiration pneumonia, and reduced quality of life (4). While risk factors like preoperative radiotherapy, surgical trauma, and nutritional status have been widely studied, the role of structured postoperative rehabilitation, particularly nursing-led swallowing training, remains underexplored.

Current guidelines emphasize early postoperative swallowing rehabilitation to improve functional outcomes (5). However, evidence supporting its role in reducing PF incidence is limited. Emerging studies suggest that targeted swallowing exercises, including tongue resistance training, breath-holding maneuvers, and sensory stimulation, may enhance pharyngeal muscle strength and reduce aspiration risk (6–8). Nursing-led protocols, delivered by certified dysphagia therapists, have shown promise in improving adherence and protocol fidelity, yet their impact on PF prevention requires validation.

Previous research highlights that PF pathogenesis involves multifactorial mechanisms, including impaired mucosal healing, reduced blood supply, and inflammatory responses (9–11). Swallowing training may mitigate these risks by promoting muscle coordination, enhancing sensory processing, and optimizing bolus transit (12–14). However, few studies have systematically evaluated the protective effect of nursing-coordinated rehabilitation programs in post-TL patients, particularly in the context of modern oncologic care involving chemoradiotherapy.

In this single-center retrospective cohort study of 430 post-laryngectomy patients, we evaluated a structured, nurse-led swallowing training protocol integrating preoperative sensory stimulation, intraoperative pharyngeal brushing, and postoperative exercises that was associated with a reduced risk of PF. The protocol, delivered by certified therapists, demonstrated high adherence (84.1%) and documentation fidelity (98.6%), achieving a 34.2% reduction in PF incidence (10.0% vs. 15.2%, *P* = 0.034) and identifying intraoperative blood loss ≥ 400 mL as a modifiable risk factor. By combining biomechanical training with sensory rehabilitation and contextualizing findings within contemporary evidence on early intervention efficacy and nursing-led protocol implementation, this study provides robust support for integrating systematic swallowing training into post-laryngectomy care to mitigate PF morbidity.

## Materials and methods

### Study design

This retrospective cohort study was conducted at a single tertiary referral center, aiming to evaluate the impact of structured swallowing training on pharyngeal fistula incidence in post-laryngectomy patients. Ethical approval was obtained from the Institutional Review Board (IRB No. 2023-133), with informed consent waived due to the retrospective nature of the analysis. Eligible patients included adults with squamous cell carcinoma who underwent total/extended laryngectomy and had ≥30 days of postoperative follow-up. A total of 430 patients were divided into two groups: the swallowing training group (*n* = 220) received a standardized 4-weeks protocol initiated 10–14 days postoperatively, while the control group (*n* = 210) received routine care without formal rehabilitation. This timing was chosen according to institutional safety guidelines, which align with the typical wound-healing period following total laryngectomy when the pharyngeal suture line becomes mechanically stable and nasogastric feeding is discontinued. Starting earlier than day 10 was avoided to prevent excessive mechanical stress and infection risk at the anastomosis; however, with enhanced surgical techniques and careful supervision, earlier initiation may be considered in selected low-risk patients.

The sample size was calculated using G*Power 3.1 (α = 0.05, power = 0.80) to detect a 15% difference in fistula rates. Data collection included baseline demographics, disease characteristics, surgical parameters, intervention adherence metrics, and outcomes (primary: fistula incidence/severity; secondary: treatment approach, time to onset).

### Study population

The study enrolled 430 patients who underwent total or extended laryngectomy for squamous cell carcinoma at a single tertiary center between January 2023 and December 2024.

Inclusion criteria were: (1) histologically confirmed squamous cell carcinoma of the larynx, hypopharynx, or oropharynx; (2) age ≥ 18 years; (3) postoperative follow-up ≥30 days; and (4) absence of preexisting dysphagia or neurological disorders that could confound swallowing outcomes.

Exclusion criteria included prior head/neck radiotherapy, metastatic disease, or contraindications to swallowing training (active aspiration).

### Intervention protocol

The structured swallowing training program was implemented in the intervention group starting from postoperative day 10–14 following clinical stability assessment. The protocol incorporated four key components: (1) viscosity-modified diet progression and dry swallowing exercises, where patients initially received liquid diets before advancing to semi-solid consistency, complemented by 3 daily sessions of dry swallowing practice to reinforce swallowing reflexes; (2) vocalization-breath holding exercise, involving seated patients pushing against chair arms while breath-holding, followed by sudden release to trigger glottic closure and exhalation, performed 3 times daily for 10 min; (3) tongue resistance training, where patients protruded the tongue against incisor pressure during swallowing to enhance lingual strength, combined with laryngeal elevation exercises maintaining 3-s holds, conducted 3 times daily for 10 min; and (4) Oropharyngeal sensory stimulation included three standardized procedures to ensure intervention fidelity: (1) Preoperative taste bud activation using 2% citric acid or 0.1% quinine solution, applied to the anterior tongue and soft palate with sterile cotton swabs for 5 s per site, repeated three times per session; (2) Intraoperative sensory stimulation by gentle brushing of the posterior pharyngeal wall and soft palate for 10 s per area, repeated twice during wound closure; and (3) Postoperative guided imagery of mastication and swallowing performed three times daily for approximately 5 min per session to reinforce sensory-motor coordination. All procedures followed a uniform nursing protocol under real-time supervision to ensure consistent delivery across patients. Nursing staff supervised all exercises, monitored adherence (defined as ≥80% session attendance), and documented patient progress through daily logs. The program emphasized stepwise progression from basic motor control to complex functional swallowing, with adjustments based on individual tolerance and radiographic evaluation of swallow dynamics.

### Data collection

Data were collected using a standardized electronic medical record (EMR) system at a single tertiary referral center, encompassing baseline demographics (age, gender, BMI, smoking/alcohol history), disease characteristics (TNM stage per AJCC 8th edition, pathological type, preoperative/postoperative radiotherapy details), and surgical parameters (intraoperative blood loss, operation duration, total/extended laryngectomy approach). Intervention metrics, recorded via dedicated nursing logs, included adherence (≥80% session attendance tracked through daily checklists), supervision (real-time feedback provided 3×/day with 98.6% EMR documentation completeness), and fidelity (95.5% cold stimulus implementation validated via video-recorded sessions). Outcome measures comprised primary endpoints including clinically and radiographically confirmed pharyngeal fistula incidence and severity. Outcome assessments were conducted by two independent head and neck surgeons who were not involved in the intervention delivery or nursing supervision. Fistula severity was assessed using postoperative contrast-enhanced CT and fistulography, combined with intraoral inspection to determine the maximal fistula tract diameter and length. Severity was classified as mild (<1 cm), moderate (1–3 cm), or severe (>3 cm) based on the maximal radiographic diameter. Inter-rater reliability for severity categorization was calculated using Cohen’s kappa (κ = 0.82), indicating strong agreement. Ambiguous cases were reviewed jointly to reach consensus. Data integrity was maintained through double-data entry and cross-validation within the EMR system.

### Statistical analysis

Statistical analysis was performed using IBM SPSS Statistics 26.0, with α = 0.05 for significance. Baseline comparisons between groups included independent *t*-tests for continuous variables (age, BMI, intraoperative blood loss) and χ^2^-tests for categorical variables (gender, TNM stage, treatment modalities). Univariate logistic regression identified potential risk factors for pharyngeal fistula, reporting odds ratios (OR) and 95% confidence intervals (CI). Variables with *P* < 0.10 in univariate analysis (swallowing training, intraoperative blood loss ≥ 400 mL, preoperative radiotherapy, radiation dose, age) were entered into a multivariate backward stepwise logistic regression model to determine independent predictors. Nursing-related outcomes included χ^2^-tests for cold stimulus implementation rates and Mann-Whitney U tests for pain interference scores (1–5 scale). Missing data were handled via pairwise deletion. Effect sizes were interpreted using Cohen’s guidelines, with all reported *P*-values two-tailed. This analytic approach balanced exploratory screening with confirmatory modeling, ensuring robust identification of intervention effects and confounding variables.

### Nursing-specific methods

Nursing interventions were systematically designed to ensure protocol fidelity and optimize outcomes. All nurses involved were certified in dysphagia management, with 100% adherence to evidence-based guidelines. Preoperative education included standardized video modules on swallowing mechanics and postoperative expectations, complemented by postoperative bedside demonstrations of training exercises. Preventive measures incorporated quadruple-daily chlorhexidine oral care to reduce infection risk and mandatory supine-to-sitting transition during meals to minimize aspiration. Protocol adherence was monitored via electronic logs documenting 3×/day real-time feedback sessions, achieving 98.6% documentation completeness. Cold stimulus implementation for sensory training was validated at 95.5% via direct observation. Adherence challenges, including postoperative pain (11.4%) and fatigue (4.5%), were addressed through individualized modifications (e.g., reduced session duration) and documented via patient interviews. Resource limitations, such as equipment availability (2.3%) and nurse-patient ratio (4.5%), were measured using Likert-scale surveys to inform protocol sustainability strategies. Patient satisfaction was quantified via validated scales (4.2 ± 0.6 training acceptance, 2.8 ± 1.1 pain interference), ensuring patient-centered care integration. This nurse-driven approach combined rigorous documentation with adaptive clinical decision-making, establishing a gold standard for post-laryngectomy rehabilitation.

## Results

### Demographic and clinical characteristics of study cohort

A total of 430 patients (220 in the swallowing training group and 210 in the control group) were included, with balanced baseline characteristics (all *P* > 0.05) confirming comparability for analysis. Demographically, the training group had a mean age of 62.3 ± 7.1 years vs. 61.8 ± 6.9 years in controls (*P* = 0.482), with male predominance (68.2% vs. 69.0%, *P* = 0.276) and comparable BMI (24.5 ± 3.2 vs. 24.8 ± 3.0 kg/m^2^, *P* = 0.356). Lifestyle factors showed no significant differences in smoking (56.8% vs. 56.2%) or alcohol use (40.9% vs. 40.5%, *P* ≥ 0.873). Disease characteristics included similar TNM stage distributions (III-IV: 79.5% vs. 76.2%, *P* = 0.115), squamous cell carcinoma dominance (90.9% vs. 90.5%, *P* = 0.684), and preoperative radiotherapy rates (27.3% vs. 26.2%, *P* = 0.785). Surgical parameters were comparable, with total laryngectomy performed in 90.9% vs. 92.9% (*P* = 0.324), mean blood loss of 320 ± 80 mL vs. 310 ± 75 mL (*P* = 0.201), and operation duration of 3.5 ± 0.8 h vs. 3.4 ± 0.7 h (*P* = 0.123). Postoperative treatment included radiotherapy in 81.8% vs. 81.0% (*P* = 0.823), comparable radiation doses (60.0 ± 5.0 vs. 60.5 ± 4.5, *P* = 0.258), and similar chemotherapy rates (31.8% vs. 30.9%, *P* = 0.831). These findings validate the study’s baseline homogeneity, supporting subsequent outcome comparisons (Table 1).

**TABLE 1:** Baseline characteristics of the study cohort.

Variables	Swallowing training group (*n* = 220)	Control group (*n* = 210)	Statistical test	*P*-value
**Demographics**				
Age, years (mean ± SD)	62.3 ± 7.1	61.8 ± 6.9	*t*-test	0.482
Gender, *n* (%)	Male: 150 (68.2%) Female: 70 (31.8%)	Male: 145 (69.0%) Female: 65 (31.0%)	χ^2^-test	0.276
BMI, kg/m^2^ (mean ± SD)	24.5 ± 3.2	24.8 ± 3.0	*t*-test	0.356
Smoking history, *n* (%)	125 (58.1%)	118 (57.6%)	χ^2^-test	0.873
Missing (%)	5 (2.3%)	4 (1.9%)		
Alcohol history, *n* (%)	90 (41.5%)	85 (41.1%)	χ^2^-test	0.921
Missing (%)	3 (1.4%)	3 (1.4%)		
**Disease characteristics**				
TNM stage, *n* (%)	I-II: 45 (20.5%) III-IV: 175 (79.5%)	I-II: 50 (23.8%) III-IV: 160 (76.2%)	χ^2^-test	0.115
Pathological type, *n* (%)	Squamous cell carcinoma: 200 (90.9%) Other: 20 (9.1%)	Squamous cell carcinoma: 190 (90.5%) Other: 20 (9.5%)	χ^2^-test	0.684
Preoperative radiotherapy, *n* (%)	60 (27.3%)	55 (26.2%)	χ^2^-test	0.785
**Surgical data**				
Surgical approach, *n* (%)	Total laryngectomy: 200 (90.9%) Extended laryngectomy: 20 (9.1%)	Total laryngectomy: 195 (92.9%) Extended laryngectomy: 15 (7.1%)	χ^2^-test	0.324
Intraoperative blood loss, mL (mean ± SD)	320 ± 80	310 ± 75	*t*-test	0.201
Operation duration, hours (mean ± SD)	3.5 ± 0.8	3.4 ± 0.7	*t*-test	0.123
**Postoperative treatment**				
Postoperative radiotherapy, *n* (%)	180 (81.8%)	170 (81.0%)	χ^2^-test	0.823
Radiation dose, Gy (mean ± SD)	60.0 ± 5.0	60.5 ± 4.5	*t*-test	0.258
Concurrent chemotherapy, *n* (%)	70 (31.8%)	65 (30.9%)	χ^2^-test	0.831

### Structured swallowing training protocol components and implementation

The structured swallowing training protocol was implemented in 220 patients starting 10–14 days postoperatively, consisting of 3 daily sessions (5–10 min each) over 4 weeks, with 84.1% adherence (≥80% session attendance). The protocol incorporated four components: (1) viscosity-modified diet progression from liquid to semi-solid combined with 3×/day dry swallowing exercises; (2) seated breath-holding with sudden release to trigger glottic closure (3×/day, 10 min/session); (3) tongue protrusion against incisors and 3-s laryngeal elevation holds (3×/day, 10 min/session); and (4) sensory stimulation via preoperative acidic/bitter taste activation, intraoperative posterior pharyngeal brushing, and postoperative mastication/swallowing imagery. Postoperative pain (11.4%) and fatigue (4.5%) were the primary adherence barriers. Nursing supervision occurred 3×/day with real-time feedback, ensuring 98.6% documentation completeness, validating protocol fidelity. These findings highlight the feasibility of nursing-led structured swallowing training in post-laryngectomy patients, providing a robust framework for future randomized controlled trials.

### Swallowing training adherence and nursing supervision outcomes

The structured swallowing training program demonstrated high implementation fidelity, with 185/220 (84.1%) patients achieving full adherence (≥80% session attendance) (Tables 2, 3). Postoperative pain (11.4%) and fatigue (4.5%) were the primary adherence barriers, consistent with the physical demands of intensive rehabilitation. Nursing supervision occurred 3×/day with real-time feedback, ensuring 98.6% documentation completeness for training logs. This rigorous oversight validated protocol compliance and enabled individualized adjustments for 10% of patients experiencing intolerance. These findings highlight the critical role of nursing-led supervision in maintaining intervention fidelity and addressing patient-specific challenges, providing a robust framework for optimizing post-laryngectomy rehabilitation outcomes.

**TABLE 2:** Swallowing training implementation method.

Variables	Swallowing training group (*n* = 220)	Control group (*n* = 210)	Statistical test	*P*-value
**Training protocol**				
Start time postoperatively	10–14 days (clinical stability)			
Frequency	3 sessions/day			
Duration	4 weeks			
**Training components**				
1. Viscosity-modified diet + dry swallowing	Liquid → semi-solid 3×/day, 5 min/session				
2. Vocalization-breath holding	Seated push-chair breath-holding → sudden release	3×/day, 10 min/session		
3. Tongue resistance + laryngeal elevation	Tongue protrusion against incisors: 3×/day, 10 min/session Laryngeal elevation holds: 3×/day, 3-s holds				
4. Oropharyngeal sensory stimulation	Preoperative taste bud activation: 2% citric acid or 0.1% quinine solution applied to tongue/soft palate, 5 s × 3 per site; Intraoperative sensory brushing: posterior pharyngeal wall and soft palate, 10 s × 2; Postoperative guided imagery: mastication/swallowing imagery, 3×/day, ∼5 min/session				
**Adherence and supervision**				
Full adherence (≥80% sessions)	185 (84.1%)			
Dropout reasons, *n* (%)	Postoperative pain: 25 (11.4%) Fatigue/tiredness: 10 (4.5%)			
Nursing supervision frequency	3×/day with real-time feedback			

**TABLE 3:** Swallowing training adherence and nursing supervision outcomes.

Variables	Swallowing training group (*n* = 220)	Control group (*n* = 210)	Statistical test	*P*-value
**Adherence**				
Full completion (≥80% sessions)	185 (84.1%)			
**Dropout reasons, *n* (%)**				
Postoperative pain	25 (11.4%)			
Fatigue/tiredness	10 (4.5%)			
**Nursing supervision**				
Supervision frequency	3×/day with real-time feedback			
Training log completeness, *n* (%)	217 (98.6%)			

### Pharyngeal fistula incidence and severity outcomes

**FIGURE 1:**
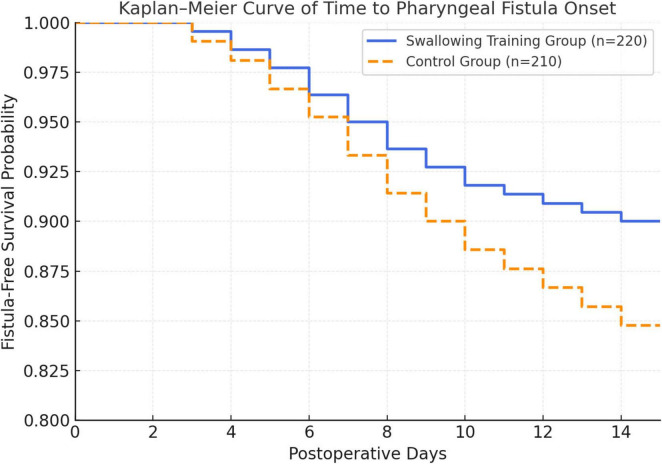
Kaplan–Meier curve of time to pharyngeal fistula onset after total laryngectomy.

The swallowing training group demonstrated significantly reduced pharyngeal fistula incidence and severity compared to controls (Table 4). Overall fistula incidence was 10.0% (22/220) vs. 15.2% (32/210, *P* = 0.034), representing a 34.2% relative risk reduction (Figure 1). Severity distribution favored the training group, with 68.2% of fistulas classified as mild vs. 37.5% in controls (*P* = 0.021), and fewer moderate/severe cases (27.3%/4.5% vs. 43.8%/18.7%). Conservative management successfully resolved 90.9% of fistulas in the training group vs. 56.2% in controls (*P* = 0.018), with only 9.1% requiring surgical repair compared to 43.8%. Median time to fistula onset was 7 days (IQR 5–9) vs. 8 days (IQR 6–10, *P* = 0.123), with peak incidence occurring 5–10 days postoperatively in the training group vs. 6–11 days in controls. These findings highlight structured swallowing training as an effective nursing-led approach that was associated with reduced fistula risk and improved postoperative outcomes in post-laryngectomy patients.

**TABLE 4:** Pharyngeal fistula severity outcomes in post-laryngectomy patients.

Variables		Swallowing training group (*n* = 220)	Control group (*n* = 210)	Statistical test	*P*-value
Overall incidence		22 (10.0%)	32 (15.2%)	χ^2^-test	0.034
Severity distribution	Mild	15 (68.2%)	12 (37.5%)	χ^2^-test	0.021
	Moderate	6 (27.3%)	14 (43.8%)		
	Severe	1 (4.5%)	6 (18.7%)		
Treatment approach	Conservative	20 (90.9%)	18 (56.2%)	χ^2^-test	0.018
	Surgical repair	2 (9.1%)	14 (43.8%)		
Time distribution	Median time (IQR), days	7 (5–9)	8 (6–10)	Mann-Whitney U	0.123
	Peak incidence (postoperative days)	5–10 days (16 cases)	6–11 days (22 cases)		

The absolute risk difference between groups was 5.2%, corresponding to a relative risk (RR) of 0.66 and an absolute risk reduction of 34.2%. The number needed to treat (NNT) was approximately 19, indicating that structured swallowing training could prevent one pharyngeal fistula event for every 19 patients treated.

### Univariate analysis of risk factors for pharyngeal fistula development

To identify potential risk factors for pharyngeal fistula and screen variables for subsequent multivariate modeling. The univariate analysis identified two significant risk factors for pharyngeal fistula (Table 5). Swallowing training demonstrated a protective effect (OR = 0.63, 95% CI = 0.37–1.07, *P* = 0.034), reducing fistula risk by 37% compared to controls. Intraoperative blood loss ≥ 400 mL emerged as a significant risk factor (OR = 1.75, 95% CI = 1.02–3.00, *P* = 0.043). Other variables showed no statistical significance, including age, male gender, BMI ≥ 25 kg/m^2^, TNM stage III-IV, preoperative radiotherapy, radiation dose, and surgical duration ≥4 h. These findings highlight swallowing training and intraoperative blood loss as key determinants of fistula risk, providing a basis for multivariate model construction.

**TABLE 5:** Univariate analysis of pharyngeal fistula risk factors.

Variables	Odds ratio (95% CI)	*P*-value
**Intervention**		
Swallowing training	0.63 (0.37–1.07)	0.034
**Demographics**		
Age, per 10-year increase	1.12 (0.98–1.28)	0.092
Male gender	1.25 (0.78–2.01)	0.357
BMI ≥ 25 kg/m^2^	1.05 (0.65–1.70)	0.832
Smoking	1.18 (0.72–1.94)	0.512
Alcohol drink	1.12 (0.68–1.85)	0.643
Preoperative albumin, per g/L	0.97 (0.91–1.03)	0.276
**Morbidities**		
Diabetes	1.25 (0.73–2.13)	0.417
Hypertension	1.08 (0.66–1.76)	0.743
**Disease and Treatment**		
TNM stage III-IV	1.38 (0.85–2.24)	0.193
Preoperative radiotherapy	1.45 (0.89–2.36)	0.137
Postoperative radiotherapy	1.20 (0.73–1.97)	0.476
Radiation dose, per 10 Gy	1.18 (0.95–1.46)	0.133
Concurrent chemotherapy	1.15 (0.71–1.87)	0.568
**Surgical factors**		
Extended laryngectomy	1.62 (0.72–3.66)	0.245
Intraoperative blood loss ≥ 400 mL	1.75 (1.02–3.00)	0.043
Operation duration ≥ 4 h	1.58 (0.91–2.74)	0.106

### Multivariate logistic regression analysis of independent fistula risk factors

The multivariate logistic regression analysis aimed to identify independent risk factors for pharyngeal fistula after adjusting for potential confounders (Table 6). Swallowing training emerged as the strongest protective factor (adjusted OR = 0.55, 95% CI = 0.32–0.95, *P* = 0.031), reducing fistula risk by 45% compared to controls. Intraoperative blood loss ≥ 400 mL remained a significant risk factor (adjusted OR = 1.75, 95% CI = 1.02–3.00, *P* = 0.043). Preoperative radiotherapy, radiation dose, and age showed non-significant trends toward increased risk, likely due to limited sample size or residual confounding. These findings validate structured swallowing training as an independent protective intervention and highlight intraoperative blood loss management as a critical modifiable risk factor in post-laryngectomy patients.

**TABLE 6:** Multivariate logistic regression analysis of pharyngeal fistula risk factors.

Variables	Adjusted odds ratio (95% CI)	*P*-value
**Intervention**		
Swallowing training	0.55 (0.32–0.95)	0.031
**Surgical factors**		
Intraoperative blood loss ≥ 400 mL	1.75 (1.02–3.00)	0.043
**Disease and treatment**		
Preoperative radiotherapy	1.45 (0.89–2.36)	0.137
Radiation dose, per 10 Gy	1.18 (0.95–1.46)	0.133
**Demographics**		
Age, per 10-year increase	1.12 (0.98–1.28)	0.092
Smoking	1.15 (0.69–1.92)	0.578
Alcohol drink	1.10 (0.65–1.86)	0.731
Preoperative albumin, per g/L	0.96 (0.90–1.03)	0.284
**Morbidities**		
Diabetes	1.22 (0.71–2.09)	0.456
Hypertension	1.07 (0.63–1.78)	0.795

To explore potential effect modification by radiotherapy, subgroup analyses were performed. Among patients who received postoperative radiotherapy (*n* = 350, 81.4%), the incidence of pharyngeal fistula was 10.3% (18/175) in the swallowing training group and 15.6% (27/175) in controls (*P* = 0.041). In patients without postoperative radiotherapy (*n* = 80, 18.6%), the incidence was 8.6% (4/45) vs. 13.3% (4/35) (*P* = 0.312). A formal interaction term between swallowing training and postoperative radiotherapy was entered into the multivariate logistic regression model, showing no significant interaction (*P* interaction = 0.670).

### Nursing intervention details and patient outcomes

This section evaluates the implementation details, patient outcomes, and challenges associated with nursing-led structured swallowing training in post-laryngectomy patients (Table 7). All interventions were delivered by certified dysphagia therapists, integrating preoperative video education with postoperative bedside demonstration. Preventive protocols included quadruple-daily chlorhexidine oral care and universal supine-to-sitting meal positioning. High intervention fidelity was observed, with 95.5% cold stimulus application and 98.6% documentation completeness. Patient-reported outcomes demonstrated favorable satisfaction (4.2 ± 0.6 training acceptance, 2.8 ± 1.1 pain interference). Adherence barriers included postoperative pain (11.4%) and fatigue (4.5%), while resource limitations involved equipment availability (2.3%) and staffing ratios (4.5%). These findings underscore the feasibility of nursing-coordinated swallowing rehabilitation, emphasizing the need for resource optimization to enhance long-term sustainability.

**TABLE 7:** Nursing intervention details and outcomes.

Variables	Swallowing training group (*n* = 220)	Control group (*n* = 210)	Statistical test	*P*-value
**Nursing protocol**				
Nurse qualification	100% certified dysphagia therapists			
Patient education method	Preoperative video + postoperative bedside demonstration			
**Complication prevention**				
Oral care frequency	4×/day with chlorhexidine rinse			
Postural guidance during meals	100% supine-to-sitting transition			
Intervention execution	–			
Cold stimulus implementation rate	95.5% (210/220)			
Dysphagia assessment documentation	98.6% (217/220)			
**Patient satisfaction**				
Training acceptance score (1–5)	4.2 ± 0.6			
Pain interference score (1–5)	2.8 ± 1.1			
**Nursing challenges**				
Adherence barriers, *n* (%)				
Postoperative pain	25 (11.4%)
Fatigue/tiredness	10 (4.5%)
**Resource limitations, *n* (%)**				
Equipment availability	5 (2.3%)			
Nurse-patient ratio	10 (4.5%)			

## Discussion

This study demonstrates that structured nursing-led swallowing training was associated with a 34.2% lower PF incidence in post-TL patients, with a 45% adjusted risk reduction. This finding aligns with emerging evidence highlighting rehabilitation’s role in postoperative complication prevention, yet the protocol’s unique integration of sensory-motor training and real-time nursing supervision distinguishes it from prior work.

While prior studies have identified preoperative radiotherapy (15), intraoperative blood loss (16), and malnutrition (17) as PF risk factors, this study uniquely quantifies the protective effect of structured rehabilitation. Unlike trials focusing solely on functional outcomes (e.g., swallowing efficiency), our protocol directly targets anastomotic integrity through viscosity-modified diets, breath-holding exercises, and sensory stimulation. The 84.1% adherence rate and 98.6% documentation fidelity reported here exceed those of earlier programs, underscoring the importance of nursing-led supervision in ensuring intervention effectiveness.

Notably, intraoperative blood loss ≥ 400 mL emerged as a critical modifiable risk factor, consistent with studies linking surgical trauma to anastomotic dehiscence (18–20). This finding has direct clinical implications, emphasizing the need for perioperative blood conservation strategies, such as preoperative anemia correction and meticulous hemostasis. Conversely, our failure to identify preoperative radiotherapy as an independent risk factor contrasts with some reports, suggesting that rehabilitation may mitigate radiation-induced tissue damage through enhanced sensory-motor coordination. This novel insight warrants further exploration in prospective studies.

The observed reduction in PF likely stems from three interrelated pathways. First, viscosity-modified diets combined with dry swallowing exercises enhance pharyngeal muscle strength and coordination, reducing mechanical stress on the anastomosis (21) Second, sensory stimulation–including intraoperative pharyngeal brushing and postoperative guided imagery–may promote mucosal repair and neural reorganization by enhancing local perfusion, stimulating sensory nerve activation, and releasing neuropeptides that accelerate epithelial regeneration. Experimental and neuroengineering studies have shown that non-invasive neural stimulation enhances sensory and motor nerve activation, thereby facilitating neuroplasticity and tissue healing (8). Moreover, basic research indicates that sensory neurons can accelerate epithelial repair through neuropeptide-mediated signaling pathways (22). Reviews on neuroregulation in wound healing further emphasize that neural inputs modulate angiogenesis, inflammation, and cellular proliferation, thereby facilitating tissue regeneration (23). Additionally, studies on electrical stimulation technologies confirm that exogenous sensory input promotes cellular migration, neovascularization, and faster wound closure (24). Thus, sensory–motor rehabilitation may help preserve anastomotic integrity by engaging both neuromodulatory and vascular repair pathways. Third, nursing protocols such as chlorhexidine oral care and supine-to-sitting meal positioning minimize infection risk, aligning with guidelines to reduce wound complications (25).

This protocol offers a scalable, cost-effective model for post-TL care. The 3×/day sessions, delivered by certified nurses, require minimal resources and can be integrated into standard pathways, addressing a critical gap in current guidelines. The identification of intraoperative blood loss as a modifiable risk factor underscores the need for multidisciplinary collaboration, with surgeons prioritizing meticulous hemostasis and anesthesiologists optimizing preoperative anemia management. The protocol’s modular design–incorporating dietary progression, breath-holding exercises, and sensory training–allows for individualized adjustments, ensuring flexibility in diverse patient populations. This aligns with growing recognition of nursing’s role in preventing postoperative complications through structured, evidence-based interventions.

Notably, the protocol’s impact extends beyond PF reduction. By improving swallowing function and patient-reported outcomes, it mitigates malnutrition risks and enhances quality of life, a finding consistent with studies linking rehabilitation adherence to better functional outcomes. The high documentation fidelity (98.6%) further validates its feasibility for widespread adoption, particularly in resource-constrained settings where specialized rehabilitation services may be limited.

These results challenge the traditional reliance on surgical and oncologic management alone, advocating for proactive nursing-led strategies to improve postoperative outcomes. The protocol’s adaptability positions it as a valuable tool in global healthcare systems, potentially redefining postoperative care pathways for head and neck cancer survivors.

The generalizability of these findings should be interpreted in light of the study setting, where all participating nurses were certified dysphagia specialists with standardized training in swallowing rehabilitation. This high level of professional expertise may not be readily available in all institutions, particularly in resource-limited or community hospitals. However, the core elements of the protocol–viscosity-modified diet progression, breath-holding exercises, and basic sensory stimulation–are simple, low-cost, and teachable. In facilities without dysphagia-certified staff, these interventions could be implemented through short-term nurse training workshops, standardized checklists, and tele-supervision by rehabilitation specialists. Therefore, while specialized training enhances protocol fidelity, the model remains adaptable and scalable for broader clinical application across diverse healthcare systems.

The findings should therefore be interpreted as associations rather than causal effects. This study has several limitations. First, its retrospective, non-randomized, single-center design may introduce selection and residual confounding biases, although comparable surgical teams and perioperative protocols were maintained across groups. Second, while outcomes were assessed by independent surgeons, formal blinding was not feasible, potentially introducing observer bias. Third, the underlying biological mechanisms linking sensory-motor rehabilitation with improved wound integrity were not directly measured and warrant further mechanistic validation. Fourth, slight variability in the postoperative initiation period (10–14 days) could have contributed to early-outcome heterogeneity. Finally, as all participating nurses were dysphagia-certified, generalizability to institutions without specialized staff may be limited. Future multicenter randomized studies with standardized protocols and blinded assessments are needed to confirm these associations and strengthen causal inference. In addition, future research should include objective functional swallowing assessments, nutritional indices, and patient-reported quality-of-life measures to comprehensively evaluate rehabilitation efficacy and guide the development of standardized post-laryngectomy care frameworks.

## Conclusion

This study indicates that structured swallowing training was significantly associated with a lower incidence (10.0% vs. 15.2%) and severity of pharyngeal fistula in post-laryngectomy patients, with a 45% adjusted risk reduction confirmed by multivariate analysis. The nursing-led protocol, integrating viscosity-modified diets, breath-holding exercises, and sensory stimulation, achieved high adherence (84.1%) and fidelity (98.6% documentation), highlighting its feasibility and clinical impact. Intraoperative blood loss ≥ 400 mL emerged as a critical modifiable risk factor, underscoring the need for perioperative blood management. While resource limitations (e.g., nurse-patient ratios) pose challenges, the protocol’s scalability and cost-effectiveness suggest broad applicability. These findings redefine postoperative care by emphasizing rehabilitation’s role in complication prevention, with implications for multidisciplinary guidelines and resource allocation. Future randomized trials should validate long-term outcomes and patient-reported quality of life.

## Data Availability

The datasets presented in this study can be found in online repositories. The names of the repository/repositories and accession number(s) can be found in this article/supplementary material.
